# Gender Differences and Work-Family Conflicts among Emergency Physicians with Intention to Leave

**DOI:** 10.1155/2018/3919147

**Published:** 2018-10-29

**Authors:** Yi-Fang Wu, Po-Chang Wang, Yi-Chuan Chen

**Affiliations:** ^1^Department of Emergency Medicine, Chang Gung Memorial Hospital, Puzi City, Chiayi, Taiwan; ^2^Department of Cardiology, Chang Gung Memorial Hospital, Puzi City, Chiayi, Taiwan

## Abstract

**Backgrounds:**

The objective of this study was to investigate the relationships among intention to leave, emergency physician clinical activities, work-family conflicts, and gender differences in emergency physicians (EPs).

**Methods:**

The survey instrument was a self-administered questionnaire containing basic demographic information and characteristics of clinical activities. The work-family conflicts were assessed by the Chinese version of the work-family conflict (WIF) scale. The questionnaires were mailed to board-certified EPs between January 2014 and August 2014. Student's* t*-test, Chi-square test, and one-way analysis of variance (ANOVA) were used to test the difference between subgroups. Logistic regression analysis was performed to determine the factors associated with intention to leave and gender differences.

**Results:**

The study included 222 respondents for analysis after exclusions. Compared with physicians not planning to leave, those planning to leave ED practice showed higher dissatisfaction with their clinical work hours (50.0% versus 31.4%, p = 0.035) and night/day shift ratio (52.9% versus 31.0%, p = 0.013) and tended to work with night/day shift ratio exceeding 40% (67.6% versus 45.7%, p = 0.019). Female physicians were more likely to leave ED practice (females versus males, 26.5% versus 10.1%, p = 0.008). A significantly higher level of WIF scale was noted in the group with intention to leave ED practice (3.7 ± 0.6 versus 3.3 ± 0.7, p = 0.001).

**Conclusions:**

Females and EPs with higher level of WIF scale were more likely to leave emergency clinical practice. Instead of the number of clinical practice hours, the satisfaction with the clinical work hours and night shift frequency were significantly associated with the intention to leave.

## 1. Introduction

The physical and emotional demands of clinical practice have made it a difficulty for emergency physicians (EPs) to maintain it as a specialty for their entire career [1–4]. Psychological distress and burnout are prevalent among EPs [5–8]. In previous studies, excessive clinical work, rotating shifts, unpredictable clinical outcomes, and work-family conflicts were the related stress factors [7, 9], which eventually led to planned reduction of clinical work and intention to leave [7, 10]. EPs have a higher probability of leaving their specialties than other specialists [11]. After leaving, the EPs either opted for retirement or pursued non-emergency clinical practice, administration, research, or teaching [12]. According to a previous study, approximately 13% of board-certified EPs turned to non-emergency clinical practice [12]. Although the proportion of female EPs has increased over the years, culture values associated with traditional gender roles still exist in Asia and North America. Females are expected to take more responsibility for household work, and although female employees spend more time on housework than male employees, the demands and expectations from clinical work are the same for both genders [13]. Females and males could have different expectations from their career and may encounter different problems in the course of their attempts to keep a balance between work and family. However, studies on how intention to leave (ITL) of EPs varies between the genders are limited.

Herein, we aimed to investigate the gender differences associated with ITL, EP clinical activities, and work-family conflicts.

## 2. Methods

### 2.1. Study Design and Population

The study consisted of a survey of EPs in Taiwan regarding the gender differences in the workload, work-family conflicts, and the intention to leave the emergency department (ED) practice within one year. The aim of this study was to survey the relationship among intentions to leave, work-family conflict, and gender differences. The survey instrument was a self-administered questionnaire containing 76 questions. Questions in this questionnaire were developed and adjusted after consultation with 20 EPs. Basic demographic information, including age, gender, marital status, and the number of children, was obtained. Characteristics of ED clinical activities, including clinical practice work hours per month, shift duration, night shifts, holiday shifts, teaching activity hours, administrative work, satisfaction with ED clinical practice, and finances, were obtained

The work-family conflicts were measured using the Chinese version of the work-family conflict scale (Carlson, Kacmar, and Williams, 2000), which had revealed good reliability and validity in the previous studies and was authorized by the Chinese author [14–16]. The scale comprises 18 items, and work-family conflict from both perspectives—work interference with family (WIF scale) and family interference with work (FIW scale)—was measured by nine items. Every three items represent three dimensions of work-family conflict, including time, strain, and behavior-based interference. Each of the items was scored on a 5-point Likert scale; 1 point indicates never and 5 points indicate always. Higher scores indicate higher levels of conflict.

The questionnaires were mailed to board-certified EPs working in ED of 27 hospitals in Taiwan, including medical centers and local hospitals, between January 2014 and August 2014. The questionnaires were collected by stamped return envelopes. Respondents who failed to answer more than 95% of the questionnaire were excluded from the survey. Consequently, the study protocol was approved by our institutional review board, and informed consent was obtained from each included EP.

The primary aim was to investigate the factors associated with ITL and gender. The secondary aim was to investigate the correlation of ITL and gender with the work-family conflict scale.

### 2.2. Statistical Analysis

All analyses were conducted using SPSS 16.0 for Windows. Student's* t*-test and Chi-square test were used to test the differences between the two groups. The difference was considered significant if p value was less than 0.05. A sample of 216 achieved a power of 80% for detecting a difference in proportions of 0.3 in the main effects of gender between the non-ITL group and the ITL group at a two-sided p value of 0.05 [17]. A logistic regression model was then developed. All variables with p value < 0.25 in the univariate analysis were entered into a multivariable logistic regression model [18]. Logistic regression models were fitted using intention to leave (ITL) and gender as a dependent variable. A p value < 0.05 was considered significant.

## 3. Results

Responses were received from 237 emergency physicians surveyed. Fifteen of the 237 survey respondents who failed to answer more than 95% of the questionnaire were excluded from the survey, leaving 222 respondents eligible for analysis. In our study, Cronbach's alpha coefficient of work-family conflict scale was 0.926.

The descriptive statistics of the study population are shown in Table 1. More than 80% of the EPs were below 45 years of age, and 87.4% of the EPs were men. Approximately 50% practiced in local hospitals, and approximately 30% of the EPs worked more than 180 hours a month in clinical ED activities. Nearly 50% of these took more than 40% of all shifts as night shifts. Overall, more than 60% planned to leave ED practice after 50 years of age, and 15.3% planned to leave ED practice within one year.

Two dichotomized variables were then created for the secondary outcome analysis on characteristics associated with EPs who planned to leave ED practice within one year (Table 2). Gender, age, and satisfaction with the work clinical hours and night shifts were significantly related to ITL within one year in ED practice. Female EPs and EPs aged ≤ 35 years were more likely to leave ED practice. EPs planning to leave ED practice were predominantly unsatisfied with their work clinical hours and night shifts and tended to work with night/day shift ratio exceeding 40%. In addition, the lower financial compensation and financial compensation bias were significantly associated with ITL. EPs who planned to retire before 45 years of age accounted for a significant percentage of EPs with ITL.

We found several gender-related differences in EPs in the present survey (Table 3). Female EPs were younger and had shorter ED practice duration than male EPs. The mean income of female EPs was less, despite the fact that there was no significant difference in ED clinical work time per month between male and female EPs; thus, a significantly higher rate of ITL was noted among female EPs. Only 35.7% female EPs had children, while 72.2% male EPs had children.

In Table 4, we present results from the analysis of relationship between work-family conflict and the intention to leave ED practice within one year. A significantly higher level of work interference with family scale (WIF scale) was noted in individuals with intention to leave ED practice. The results showed a higher score on time-based and strain-based interference in reviewing the three dimensions of WIF scale. As in WIF scale, a significantly higher level of the time-based interference was noted in FIW scale as well. The FIW conflict was significantly lower in the group of clinical work hours less than 120 hours per month (2.4 ± 0.8 versus 2.8 ± 0.6, p < 0.05). There were no gender differences in work-family conflict in ED physicians (Table 5). WIF conflict (2.6 ± 1.0 versus 3.4 ± 0.7, p < 0.05) and FIW conflict (2.1 ± 0.6 versus 2.8 ± 0.7, p < 0.01) were significantly lower in physicians aged more than 55 years. Both WIF conflict (p < 0.001) and FIW conflict (p < 0.01) were significantly higher when EPs felt unsatisfied with clinical work hours and financial compensation fairness. WIF conflict was higher when EPs were unsatisfied with night shift frequency (p = 0.001).

Table 6 summarizes the results of logistic regression for factors associated with intention to leave. After entering all the variables with p value < 0.25 in Tables 2 and 4, retirement preference before 45 years of age (adjusted odds ratio [OR], 10.51; 95% confidence interval [CI]: 3.99-27.68), night/day shift ratio >40% (OR, 2.70; 95% CI: 1.11-6.57), and time-based family interference with work (FIW) (OR, 4.56; 95% CI: 1.68-12.37) were associated with ITL.  Table 7 summarizes the results of logistic regression for factors associated with gender differences. Age below 35 years (OR, 3.17; 95% CI: 1.10-9.11), shift length of 12 hours (OR, 5.67; 95% CI: 1.96-16.39), and EPs with children (OR, 0.18; 95% CI: 0.06-0.52) were associated with gender differences.

## 4. Discussion

### 4.1. The Attrition and ITL of Emergency Physicians

The ITL rate of EPs ranges from 12% to 21.4% per year [7, 19]. Frequent ITL from the profession may eventually cause increased attrition of EPs. The attrition rate within 2 years since training graduation was 6.5%, and the estimated EP attrition rate was 1.7% per year in USA [12]. Similar results were noted in Taiwan; the annual attrition rate was 1.83% [11].

Our study showed that 15.3% of the respondents had ITL within one year, and 34.2% planned to retire before 50 years of age. EPs in Taiwan in our study were younger compared to those in previous studies conducted in USA. The mean age of our respondents was 39.0 ± 7.6 years; 66% of our respondents were aged less than 40 years. The mean age was 42.5 ± 6.0 years, and 34% of EPs were less than 39 years of age in the study of Lynnette Doan-Wiggins et al. in 1995 [20]. The mean age of EPs was 49.9 ± 10.5 years in a study conducted in 2004 in USA [21]. Younger mean age of EPs in our present study than other studies may result from the shorter duration, since we had board-certified EPs in Taiwan (the American Board of Emergency Medicine was incorporated in 1976 and Taiwan Society of Emergency Medicine in 1994). Another explanation is that, compared with surgeons and radiologists in Taiwan, the nonfailure rate for EPs after 10 years of practice is significantly decreased [11]. The trend of decrease in nonfailure rate was not noted in other regions, which showed that the cumulative and annual attrition rate did not rise rapidly until 30 years since training graduation [12]; these findings may indicate a shorter career life of EPs in Taiwan.

In our study, EPs below 35 years of age were more likely to leave ED practice; this is in agreement with the finding of highest annualized attrition rates of EPs for the first 5 years after graduation [12]; however, it is not consistent with the findings of the previous SESMAT study, which showed that young age was not linked with ITL [7]. We found that EPs who planned to retire before 45 years of age accounted for a significantly high percentage in the group with ITL within one year (41.2% versus 8.5%, p < 0.001); this revealed that EPs who prefer to retire earlier were also bearing a high risk of job turnover.

Some factors related to ITL of EPs have been discussed, including teamwork, burnout, and job satisfaction. Low quality of teamwork caused a 4-fold increase in ITL, while burnout doubled the risk of ITL for EPs [7]. EPs were found to have the highest rate of burnout (65%) compared with physicians in general, and 60% of EPs had moderate-to-high burnout scores [6, 8, 22, 23]. Physicians planning to leave practice were more likely to report feeling of burnout and have lower practice satisfaction [6, 19]. Despite the high level of burnout, job satisfaction was also reportedly high among EPs in previous studies. More than three-fourths of EPs stated that emergency medicine has met or exceeded their career expectations [21, 22]. However, the situation varied in different countries and regions. Chinese EPs had only a moderate level of job satisfaction and were generally not satisfied with their work [5], and job satisfaction was an influencing factor of turnover intention [24].

Schedule flexibility is a key determinant of choosing a career in emergency medicine and ongoing career satisfaction [25]. Excessive clinical workload and night shifts were associated with burnout [6, 20, 22, 26]. Higher levels of burnout were noted among EPs whose practices were primarily clinical, and the levels of burnout peaked at 13–15 shifts per month [6]. Physicians who do not have enough time for personal life are three times as likely to report low career satisfaction and twice as likely to experience burnout [21]. Women physicians are more likely to alter their job responsibilities for the benefit of their families and children, with the most common adjustment being reduced working hours [27].

Rotating shifts, including night shifts and weekend shifts, allow for the around-the-clock operation of the emergency room. However, EPs working night shifts were found to have decreased sleep, negative mood states, and poor performance compared to EPs working day shifts [28–30]. Working five consecutive night shifts results in a substantial decline of cognitive performance in EPs [31]. Despite the negative effects of excessive work hours and night shifts on EPs, clinical work hours and night shifts were not found to be related to ITL in French SESMAT [7]. Our study revealed that EPs consider leaving their job when night shifts account for over 40% of their total shifts. Strategies for enhancing adaptation to night shifts, such as clockwise shift rotations [32–34], rotating shifts after 2-week periods, and an average of 2 days off per week, were suggested [34]. Interventions for enhancing the adaptation of EPs to night shifts and rotations need more research.

Fair and high financial compensation were associated with high career satisfaction, and dissatisfaction with pay was significantly related to ITL [7, 21]. The result was also noted in our study group.

EPs who were married and had children were less likely to leave their job; the trend was in agreement with a previous report [7]. The possible explanation may be the impacts of financial obligations associated with career alterations on family and children, which made EPs continue their practice. Other factors linked to ITL include worries about making mistakes, absence of continuing education, harassment by superiors, and relationships with administration [7].

### 4.2. The Gender Differences

Male EPs seemed to remain in their specialties longer than female EPs [11]. In our study, female EPs had shorter ED practice duration, and more than 70% were less than 35 years. Female EPs in our study were younger than male EPs in Taiwan and their female counterparts in USA [35]. The attrition rate of female EPs remained unclear; however, younger age may be associated with a higher attrition rate and a shorter career span in them. Compared to male EPs, a significantly higher percentage of female EPs reported ITL (32.1% versus 12.9%, p < 0.01) in our study. The gender differences on ITL of EPs were not noted in previous studies conducted in other regions [7]. The majority of the female EPs were without children, which was the opposite condition compared to male group. The result is consistent with that of a previous study [35]. Taking care of family and young children is still mostly a woman's responsibility in Asia; this may force female EPs to leave or alter their work before they became as experienced as male EPs.

The gender differences on burnout score varied in different studies [7]. Career satisfaction did not differ between male and female EPs [6, 20, 21]. Schedule flexibility, fairness in financial compensation, and opportunities for career advancement were key factors in career satisfaction among female EPs [35].

### 4.3. The Work-Family Interference in Emergency Physicians

Conflicts occurred when expectations and demands at either work or in the family were not met. High work-family conflict is a major factor associated with burnout of EPs and remains the highest risk factor of burnout for female EPs [7, 36]. In addition, higher levels of work-family conflict were associated with higher odds of preferring partial or full retirement within 10 years in both male and female EPs [37], and work-family conflict significantly was related to ITL [7, 22, 38, 39]. Studies on the relationship between work-family conflict and ITL of EPs are limited. In our survey, a significant high level of work interference with family was noted in EPs with ITL. The result is consistent with previous research, which showed that the work-to-family conflict (but not family-to-work conflict) was positively related to ITL [38, 39]. This could be explained by family boundaries being more permeable than work boundaries, which had made the work-to-family conflict more prevalent [40].

In reviewing the three dimensions of work-family conflict, time-based interference was significantly higher on both WIF scale and FIW scale on ITL of all EPs and on WIF scale of female EPs. Employees with more work hours a week had higher work-family conflict (WFC) and higher end-of-workday strain [41]. Higher WFC was noted in EPs who were unsatisfied with clinical working hours and night shifts. Employees who were offered greater work-time control and flexibility had lower odds of turnover [42].

Previous studies revealed conflicting results about gender differences on work-family conflict; some showed higher level of WFC in female physicians [43–45], while some reported no significant differences between male and female EPs [38, 46]. Our survey showed higher scores for female EPs in both WIF scale and FIW scale; however, the result is not significant. However, considering younger age, shortened practice duration, lower rates of having children, and higher ITL in female EPs, we should pay attention to the possibility of female EPs who experienced more work-family conflicts, failed to construct coping strategies, and left clinical practice. Further studies focusing on these issues are warranted.

### 4.4. Limitation

Several limitations of the study shall be acknowledged. First, the cross-sectional design of the current study has resulted in the low possibility to draw causal relations among work-family conflict, gender difference, and intention to leave. Second, as in other questionnaire surveys, response bias could exist. It is possible that those with stronger opinions and those with higher motivations responded and completed the survey.

## 5. Conclusion

Our studies showed that work schedule satisfaction, including clinical work hours and night/day shift ratio, was strongly related to ITL. Females and EPs with higher level of work interference with family scale were more likely to have ITL. On reviewing the work-family conflict three-dimensionally, time-based interference was significantly higher on both WIF scale and FIW scale on ITL of EPs and on WIF scale of female EPs. For both male and female EPs, time remains a critical and important issue when trying to construct strategies to maintain ED clinical practice as an entire career.

Further studies should focus on developing strategies on the schedule arrangement to minimize the work stress and work-family conflicts for both females and males.

## Figures and Tables

**Table 1:** The characteristics of the study population.

**Descriptive statistics**	Number of respondents	%
	N = 222	
**Age group**		
≤30	16	7.20%
31–35	70	31.50%
36–40	61	27.50%
41–45	37	16.70%
46–50	18	8.10%
>50	20	9.00%
Mean ± SD	39.0 ± 7.6 years	
**Gender**		
Male	194	87.40%
Female	28	12.60%
**ED practice hospital level**		
Medical center	108	48.65%
Local hospital	114	51.35%
**Shift length**		
8 h	31	14%
12 h	110	49.50%
Mixed shifts	76	34.20%
Others	5	2.30%
**Clinical work time per month (hours)**		
≤120	19	8.60%
121–140	21	9.50%
141–160	49	22.10%
161–180	67	30.20%
181–200	50	22.50%
>200	16	7.20%
**Night/day shift ratio**		
Day shift only	16	7.20%
<20%	29	13.10%
21–40%	68	30.60%
41–60%	75	33.80%
61–70%	15	6.80%
>70%	19	8.60%
**Weekend shifts per month**		
1–3	33	14.90%
4–6	178	80.20%
>6	11	5.00%
**Financial compensation per month (10,000 NTD)**		
<30	100	45.00%
30–40	115	51.80%
>40	7	3.20%
**Marital status**		
Married	178	80.18%
Others	44	19.82%
Children		
Yes	150	67.60%
No	72	32.40%
**Intention to leave within one year**		
Yes	34	15.30%
No	188	84.70%
**Retirement age preference (years old)**		
<45	30	13.50%
46–50	46	20.70%
51–55	63	28.40%
>55	83	37.40%

SD: standard deviation; ED: emergency department; NTD: New Taiwan dollars.

**Table 2:** Characteristics associated with emergency physicians who planned to leave emergency department (ED) practice within one year.

	**Intention to leave ED practice within one year**				p
	NO		Yes		
	N.	%	N	%	
**Gender**					
Male	169	89.90%	25	73.50%	0.008
Female	19	10.10%	9	26.50%	
**ED practice hospital level**					
Medical center	91	48.40%	17	50.00%	0.86
Local hospital	97	51.60%	17	50.00%	
**Age**					
Mean ± SD	39.6 ± 7.6		36.0 ± 7.0		0.012
Age group (years)					
≤35	64	34.00%	22	64.70%	0.001
>35	124	66.00%	12	35.30%	
**Clinical work time per month (hours)**					
>180	55	29.30%	11	32.40%	0.72
Less than 180	133	70.70%	23	67.60%	
**Satisfaction with clinical work hours**					
Yes	129	68.60%	17	50.00%	0.035
No	59	31.40%	17	50.00%	
**Satisfaction with night shift amount**					
Yes	127	69.00%	16	47.10%	0.013
No	57	31.00%	18	52.90%	
**Night/day shift ratio**					
≤40%	102	54.30%	11	32.40%	0.019
>40%	86	45.70%	23	67.60%	
**Weekend shift**					
1–4 shifts	88	46.80%	16	48.50%	0.86
≥5 shifts	100	53.20%	17	51.50%	
**Shift length**					
12 h	91	48.40%	19	55.90%	0.42
others	97	51.60%	15	44.10%	
**Financial compensation per month (NTD)**					
≤300,000	76	40.40%	22	64.70%	0.009
>300,000	112	59.60%	12	35.30%	
**Financial compensation fairness**					
Yes	104	55.60%	12	35.30%	0.029
No	83	44.40%	22	64.70%	
**Administration work**					
Yes	43	23.20%	9	26.50%	0.68
No	142	76.80%	25	73.50%	
**Teaching activity per month (hours)**					
≥40	11	5.90%	3	8.80%	0.46
<40	177	94.10%	31	91.20%	
**Retirement age preference (years)**					
≤45	16	8.50%	14	41.20%	<0.001
46–50	41	21.80%	5	14.70%	
51–55	53	28.20%	10	29.40%	
>55	78	41.50%	5	14.70%	
**Marital status**					
Married	155	82.40%	23	67.60%	0.046
Others	33	17.60%	11	32.40%	
**Children**					
Yes	133	70.70%	17	50.00%	0.017
No	55	29.30%	17	50.00%	

ED: emergency department; SD: standard deviation; NTD: New Taiwan dollars.

**Table 3:** Gender differences in emergency physicians.

	**Male**		**Female**		
	Number	%	Number	%	p
**ED practice hospital level**					
Medical center	92	47.40%	16	57.10%	0.34
Local hospital	102	52.60%	12	42.90%	
**Age**					
Mean ± SD	39.6 ± 7.7		34.6 ± 5.3		0.001
**Age group (years old)**					
≤35	66	34.00%	20	71.40%	<0.001
>35	128	66.00%	8	28.60%	
**ED practice length (years)**					
Mean ± SD	7.4 ± 6.1		4.2 ± 3.8		0.001
**Clinical work time per month (hours)**					
Mean ± SD	165.4 ± 30.6		157.3 ± 26.7		0.18
**Satisfaction with clinical work time**					
Yes	126	64.90%	20	71.40%	0.50
No	68	35.10%	8	28.60%	
**Satisfaction with night shift amount**					
Yes	128	66.70%	15	57.70%	0.37
No	64	33.30%	11	42.30%	
**night/day shift ratio**					
≤40%	98	50.50%	15	53.60%	0.76
>40%	96	49.50%	13	46.40%	
**Weekend shift**					
1-4 shifts	92	47.40%	12	44.40%	0.77
≥5 shifts	102	52.60%	15	55.60%	
**Shift length**					
12 h	91	46.90%	19	67.90%	0.038
others	103	53.10%	9	32.10%	
**Financial compensation per month (10,000 NTD)**					
Mean ± SD	31.0 ± 7.0		26.0 ± 7.6		0.001
**Financial compensation fairness**					
Yes	98	50.80%	18	64.30%	0.18
No	95	49.20%	10	35.70%	
**intention to leave within one year**					
Yes	25	12.90%	9	32.10%	0.008
No	169	87.10%	19	67.90%	
**Administration work**					
Yes	48	25.10%	4	14.30%	0.24
No	143	74.90%	24	85.70%	
**Marital status**					
Married	158	81.40%	20	71.40%	0.21
Others	36	18.60%	8	28.60%	
**Children**					
Yes	140	72.20%	10	35.70%	<0.001
No	54	27.80%	18	64.30%	

ED: emergency department; SD: standard deviation; NTD: New Taiwan dollars.

**Table 4:** Relationship between work–family conflict and intention to leave emergency department (ED) practice within one year.

Work–family conflict		Intention to leave within one year		p
		Yes	No	
		Mean ± SD	Mean ± SD	
Work interference with family (WIF)		3.7 ± 0.6	3.3 ± 0.7	0.001
	Time based	4.0 ± 0.8	3.5 ± 0.8	0.003
	Strain based	3.9 ± 0.7	3.3 ± 0.9	<0.001
	Behavior based	3.3 ± 0.8	3.1 ± 0.8	0.11

Family interference with work (FIW)		3.0 ± 0.6	2.7 ± 0.7	0.06
	Time based	3.2 ± 0.9	2.7 ± 0.8	0.004
	Strain based	2.8 ± 0.9	2.6 ± 0.8	0.15
	Behavior based	2.9 ± 0.8	2.8 ± 0.8	0.70

SD: standard deviation.

**Table 5:** Gender and work–family conflict in emergency physicians.

Work–family conflict		Male	Female	p
		Mean ± SD	Mean ± SD	
Work interference with family (WIF)		3.3 ± 0.7	3.5 ± 0.6	0.25
	Time based	3.5 ± 0.9	3.9 ± 0.6	0.012
	Strain based	3.4 ± 0.9	3.7 ± 0.8	0.11
	Behavior based	3.1 ± 0.8	3.0 ± 0.8	0.4

Family interference with work (FIW)		2.7 ± 0.7	2.8 ± 0.6	0.90
	Time based	2.8 ± 0.8	2.9 ± 0.8	0.62
	Strain based	2.6 ± 0.8	2.8 ± 0.8	0.32
	Behavior based	2.9 ± 0.8	2.7 ± 0.7	0.34

SD: standard deviation.

**Table 6:** Logistic regression analysis of factors associated with intention to leave (ITL).

**Variables**	**Adjusted odds ratio**	**95**%** CI**	**P**
**Retirement age preference (years) ≤45**	10.51	3.99-27.68	<0.001
**Night/day shift ratio >40**%	2.70	1.11-6.57	0.029
**Family interference with work (FIW), time based**	4.56	1.68-12.37	0.003

CI: confidence interval.

**Table 7:** Logistic regression analysis of factors associated with gender differences.

**Variables**	**Adjusted odds ratio**	**95**%** CI**	**P**
**Age group (years old) ≤35**	3.17	1.10-9.11	0.032
**Shift length 12 h**	5.67	1.96-16.39	0.001
**With Children **	0.18	0.06-0.52	0.001

CI: confidence interval.

## Data Availability

Due to the sensitive nature of the questions asked in this study, survey respondents were assured that raw data would remain confidential and would not be shared. Data is not available/the data that has been used is confidential.
